# Recombinant Lz-8 from *Ganoderma lucidum* induces endoplasmic reticulum stress-mediated autophagic cell death in SGC-7901 human gastric cancer cells

**DOI:** 10.3892/or.2011.1593

**Published:** 2011-12-14

**Authors:** CHONGYANG LIANG, HONGRUI LI, HUI ZHOU, SHUQIN ZHANG, ZHIYI LIU, QIULI ZHOU, FEI SUN

**Affiliations:** Institute of Frontier Medical Science of Jilin University, Changchun 130021, P.R. China

**Keywords:** *Ganoderma lucidum*, rLz-8, autophagy, endoplasmic reticulum, ER-associated degradation

## Abstract

In Asia, the mushroom of the fungus *Ganoderma lucidum* has been widely used as a traditional medicine for the past two millennia. The aim of this study was to investigate the anticancer activity of recombinant Lz-8 (rLz-8), a protein belonging to a family of fungal immunomodulatory proteins. We report that rLz-8 induces endoplasmic reticulum (ER) stress-mediated autophagic cell death in the human gastric cancer cell line SGC-7901. Our results show that rLz-8 induces autophagic cell death by aggregating in the ER, triggering ER stress and the ATF4-CHOP pathway. A foreign protein, in the ER rLz-8 causes the activation of the ubiquitine/proteasome ER-associated degradation (ERAD) system. The autophagic arm of this system is then overstimulated by an excessive abundance of rLz-8 and causes the cell’s death through an over-autophagic response. We also found that caspase inhibitors do not prevent rLz-8-induced cell death, and therefore the autophagic response induced by rLz-8 is independent of caspase activation.

## Introduction

Proteins must be folded into proper conformations in order to carry out their cellular functions (1). The endoplasmic reticulum (ER) is the site of synthesis and folding of proteins destined for either secretion or locations in the cell membrane, Golgi apparatus, lysosomes and elsewhere (2). However, when protein folding in the ER is impaired due to various physiological and pathological conditions, unfolded or misfolded proteins can threaten cell survival. The unfolded protein response (UPR) of the cell stresses the ER and triggers an ER quality-control system that recognizes and removes nascent proteins that fail to fold or assemble properly (3,4).

The UPR system is composed of two ER-associated degradation (ERAD) systems, the ubiquitin/proteasome and the autophagy/lysosome (5). Fujita *et al* (5) used dystrophy-associated fer-1-like protein (dysferlin) degradation to investigate two ERAD models and found that when misfolded dysferlin aggregated on the ER membrane excessively, the cell chose the autophagy/lysosome ERAD system rather than the ubiquitin/proteasome (6,7). It is known that autophagy is a self-digestion process that degrades intracellular structures in response to stresses, whose purpose is cell survival. However, if autophagy was prolonged, it leads to cell death (8). Therefore, the autophagy/lysosome ERAD system may open a door for purposively causing cell death.

In recent years, the relationship between the intracellular aggregation of unfolded or misfolded proteins and ER stress has been intensively examined (9). However, there are few reports in the cell biological literature regarding ER stress and autophagy induced by a heterologous protein. In this study, we found that recombinant Lz-8 (rLz-8), a protein from the fruiting body of the bracket fungus *Ganoderma lucidum*, aggregated on the ER of human gastric cancer SGC-7901 cells. Accumulation of rLz-8 induced ER stress and autophagy, leading to massive death of cells, independent of caspase activity. The aims of this study were to determine how cells deal with a heterologous protein such as rLz-8 that accumulates on the ER, and the role of ER stress and autophagy in triggering cell death.

*Ganoderma lucidum*, a popular medicinal mushroom, has been widely used in traditional Chinese medicine in many Asian countries during the past two millennia. It has been reported to be effective in modulating immune functions, inhibiting tumor cell growth and allergies (10) and in the treatment of chronic hepatitis, hypertension, and hyperglycemia (11). *G. lucidum* polysaccharide and triterperoid were the major bioactive substances until immunomodulatory proteins, Lz-8, was isolated and purified from the mycelia of *G. lucidum* in 1989 (12–14). Previously, we demonstrated the crystal structure of Lz-8 which was a noncovalently linked homodimer with an apparent molecular weight of 24 kDa. Each monomer consists of 110 aa residues with an acetylated N terminus and a molecular mass of 12 kDa (15). Liao *et al* also reported that reFIP-gts, another immunomodulatory protein from the closely related Ganoderma tsugae, inhibited the growth of A549 cancer cells significantly and selectively (16). However, until now there have been no studies demonstrating how Lz-8 induces cell death and the mechanisms involved in this process.

Herein we report for the first time that an excessive heterologous protein aggregation of rLz-8 from *Ganoderma lucidum* on the ER of human cancer cells induces autophagy-dependent cell death, not a caspase-dependent cell death or apoptosis, and provides a novel strategy for cancer treatment.

## Materials and methods

### Recombinant plasmid construction and Pichia pastoris transformation

The total DNA of *G. lucidum* was extracted as described by Al-Samarrai and Schmid (17). The rLz-8 gene was amplified from the total DNA sample by PCR, and *Sna*BI and *Eco*RI restriction sites were designed for flanking the PCR product at the 5′- and 3′-termini, respectively. To build the expression plasmid, the PCR fragment was cloned into the *Sna*BI/*Eco*RI site of pPIC9K (Invitrogen, CA, USA) and transformed into *Pichia pastoris* Gs115 (Mut^+^; Invitrogen).

### Media and culture conditions for rLz-8 expression

The *P. pastoris* transformants were cultured in a 100 ml flask containing 1000 ml buffered minimal glycerol-rich yeast (BMGY) medium, supplemented with 1% (v/v) glycerol as a carbon source and 200 μg/ml G418 (Geneticin) as a selection pressure. Cells were grown at 28˚C and shaken at 300 rpm until an OD_600_ value reached 15 making a 10-fold dilution. The cells were then harvested by centrifugation at 3000 × g and 4˚C for 5 min. BMGY was replaced by buffered minimal methanol-rich yeast (BMMY) medium containing 0.5% (v/v) methanol. Methanol was used to induce the AOXI promoter. BMGY and BMMY media were also prepared according to the manufacturer's instructions (Invitrogen). After 48 h of induction, the supernatant was collected by centrifugation at 4˚C and 12,000 × g for 20 min. rLz-8 protein was purified by a nickel affinity column Ni-sepharose (GE Lifescience, USA) and eluted by a gradient of 30–100 mM imidazol.

### Cell culture and materials

Cells of the human gastric cancer cell line SGC7901 were obtained from the Institute of Biochemistry and Cell Biology (Shanghai, China). Cells were grown in Dulbecco's modified Eagle's medium (DMEM; Invitrogen) supplemented with 2 mM L-glutamine, 100 U/ml penicillin, 100 μg/ml streptomycin, and 10% fetal bovine serum (FBS) at 37˚C in a humidified atmosphere of 95% air and 5% CO_2_. Anti-ATF4 antibody was purchased from Proteintech Group (PTG, USA). Anti-CHOP, Anti-LC3, Anti-GAPDH antibodies and secondary antibody were purchased from Santa Cruz Biotechnology (Santa Cruz, Canada). Tm, TG and 3-MA were obtained from Sigma-Aldrich (USA).

### Electron microscopy

SGC7901 cells were double fixed in PBS-buffered glutaraldehyde (2.5%) and osmium tetroxide (1%), dehydrated, and embedded using the EMBed-812 kit (EMS, USA) according to the manufacturer's instructions. Ultrathin sections (70 nm) were made and double stained with uranyl acetate and lead citrate, and viewed in a transmission electron microscope (Hitachi H-7500, Japan).

### Western blotting

Cells were washed with PBS and lysed in Lysis-M Reagent supplemented with complete mini-protease inhibitor cocktail tablets (Roche, Indianapolis, IN, USA). The lysates were then incubated for 5 min at room temperature with gentle shaking, and centrifuged at 14,000 × g for 10 min. Equal amounts of protein were subjected to 12% sodium dodecyl sulfate polyacrylamide gel electrophoresis and then transferred to polyvinylidene difluoride membranes (Millipore, USA). The membranes after blocking in 5% skimmed milk powder in TBS were incubated with each primary antibody followed by a peroxidase conjugated anti-rabbit secondary antibody. The corresponding bands were detected using an ECL Advance kit (GE Biosciences, USA).

### Real-time quantitative PCR

The total RNA was isolated using Trizol (Invitrogen) from cells after treatment with or without rLz-8 for the indicated times. cDNA was synthesized using a reverse transcription system (Takara, Japan). The real-time PCR was performed with the use of LineGene 3320 Bioanalyzer (Bioer, China) following the manufacturer's instructions. SYBR Green I kit (Takara) was used. PCR conditions were 95˚C for 30 sec followed by 40 cycles of 95˚C for 5 sec and 60˚C for 20 sec. Bioer 3320 software was used to analyze the standards and to carry out the quantification. The relative quantity of each mRNA was normalized to the relative quantity of GAPDH mRNA. ATF4 sense primer: 5′-TCTCC AGCGACAAGGCT AAG-3′; ATF4 anti-sense primer: 5′-TACCCAACAGGGCATC CAAG-3′; CHOP sense primer: 5′-GAACCAGGAAACGGAA ACAG-3′; CHOP anti-sense primer: 5′-CATTCACCATTCGG TCAATCA-3′; LC3 sense primer: 5′-AGCAGCATCCAACC AAAATC-3′; LC3 anti-sense primer: 5′-CTGTGTCCGTTCA CCAACAG-3′; GAPDN sense primer: 5′-GCACCGTCAAGG CTGAGAAC-3′; GAPDH anti-sense primer: 5′-ATGGTGGTG AAGACGCCAGT-3′.

### Cell proliferation assays

The inhibition of cell proliferation and viability in SGC7901 cells was determined using the WST-1 (Roche) assay. Cells were placed at 8,000 per well in 96-well plates in their respective growth medium with FBS reduced to 5%. The cells were allowed to grow for 24 h and then treated with different drugs. After 24 h, WST-1 reagent was added to the plates according to manufacturer's protocol, and absorbance was read at 450 nm with an ELISA reader (Tecan).

### Confocal microscopy

ERtracker, mitotracker and lysotracker (Invitrogen) was used for the assessment of cellular lysosomal content, according to the manufacturer's instructions. Briefly, cells were cultured on 24-well plates and treated with rLz-8- fluorescein isothiocyanate (FITC) or rLz-8 (5 μg/ml). After several time courses, cells were incubated with 1 μM ER tracker, 300 nM mitotracker, or 75 nM Lysotracker for 45 min at 37˚C in 5% CO_2_, and nuclear stained with 1 μg/ml Hoechst33342 (Sigma, USA) for 15 min. Cell were washed thrice with fresh medium and were visualized using an Olympus laser scanning confocal fluorescence microscope fitted with the appropriate filters. The results of Lysotracker fluorescence were selected, five fields of view (512×512 pixel) per section, and fluorescence images were analyzed using ImageJ software (NIH).

### Measurement of caspase activity

Caspase-3 activity in SGC7901 cells was measured with a NucView 488 Caspase-3 Assay Kit for live cells according to the manufacturer's instructions (Biotium, Hayward, CA, USA). Briefly, the cells were grown in 24-well plates at a density of 1×10^5^ cells per well. After rLz-8 treatment for 24 h, cells were incubated with 3 μm NucView 488 Caspase-3 substrate in medium for 30 min. Cells (200/group) were visualized using a fluorescence microscope fitted with the appropriate filters. For the inhibition of caspases, cells were grown in 96-well plates. After 24 h, the cells were pre-incubated with 50 μM z-VAD-fmk (a pan-caspase inhibitor) for 30 min and then treated with rLz-8 for 48 h. The results were measured with the WST-1 assay.

### Cell cycle analysis

Cells were placed into 6-well plates at a density of 5×10^5^ cells per well and incubated with media or rLz-8 at 5 μg/ml for the indicated times. Cells were harvested by centrifugation at appropriate times and fixed with 70% ethanol at 4˚C overnight. After fixation, cells were washed by centrifugation with PBS and resuspended in 1 ml PI staining buffer (0.1% Triton X-100, 100 μg/ml RNase A, 50 μg/ml PI in PBS) at 37˚C for 30 min. The cell cycle phase was analyzed using a flow cytometer (Becton Dickinson, USA) with Cellquest software.

### Assessment of apoptosis

The effects of rLz-8 on nuclear morphology in cells were examined by staining with Hoechst 33342 (Sigma). In brief, cells (1×10^5^ cells/well; 24 wells) were incubated with rLz-8 (5 μg/ml) in the growth medium for 24 h at 37˚C. The cells were stained with Hoechst 33342 (1 μg/ml) for 15 min and washed twice with PBS. The images were observed under a fluorescence microscope (Olympus X71, Japan).

Evaluation of apoptotic cell death was performed with the Annexin V-FITC Apoptosis Detection kit (Bipec Biopharma, USA) according to the manufacturer's recommendations. In brief, after the treatment, cells were washed twice with cold PBS and collected by centrifugation. The pellet was resuspended in 1X binding buffer and stained with FITC-labeled Annexin V (5 μl) for 15 min at 4–8˚C in the dark. PI (10 μl) was then added for 5 min at 4–8˚C in the dark. Cells were analyzed using a flow cytometer (Becton Dickinson) with Cellquest software.

### Mitochondrial membrane potential

Mitochondrial membrane potential was examined by JC-1, according to the manufacturer's instructions. Briefly, cells were placed on 6-well plates and treated with rLz-8 (5 μg/ml). After 24 h, cells were incubated with 10 μg/ml JC-1 for 30 min at 37˚C in 5% CO_2_ and then analyzed using a flow cytometer.

### Small interfering RNA (siRNA)

The ATF4 siRNA or CHOP siRNA duplexes used in this study were purchased from Santa Cruz Biotechnology. SGC7901 cells, plated 1 day earlier on 6-well culture dishes to achieve 50–60% confluence, were transfected with siRNAs (80 pM) by using 6 μl of Lipofectmine 2000 (Invitrogen). After 24 h of transfection, cells were treated with rLz-8 for up to 24 h.

### Statistical analyses

Statistical analysis was conducted using Student's t-test and one-way ANOVA. The data were presented as mean ± standard error (SE). Experiments were performed at least three times. Results were considered to be statistically significant at P<0.05.

## Results

### rLz-8 inhibits cell growth and induces cell death in human gastric cancer cell line SGC-7901

**Figure 1:**
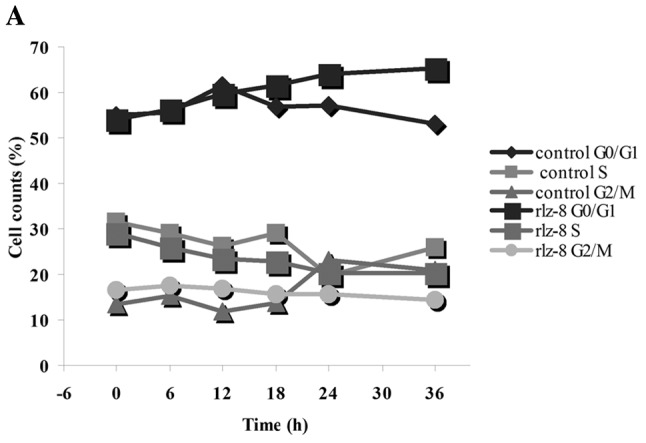

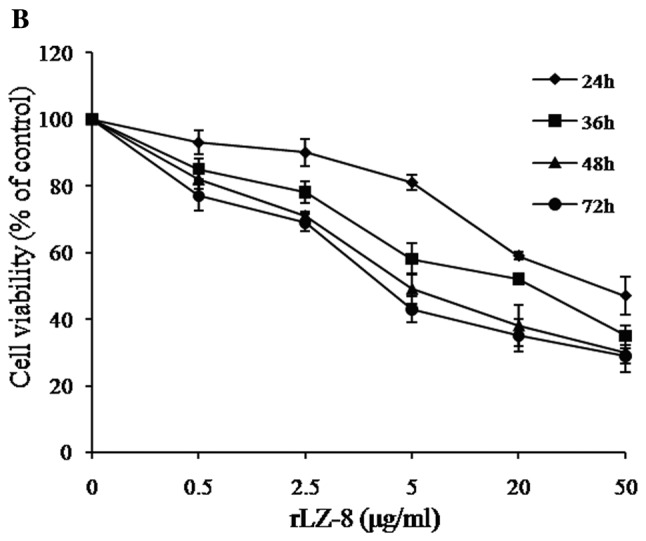
Inhibition of cell growth of SGC7901 cells treated with rLz-8. (A) Effect of rLZ-8 on cell cycle progression in SGC7901. Cells were treated with or without rLz-8 (5 μg/ml) for several time coures (0, 6, 12, 18, 24 and 36 h). Cells were stained with propidium iodide and detected using a flow cytometer. (B) rLz-8 inhibits cell proliferation. SGC7901 cells treated with a range of rLz-8 concentrations (0.5 to 50 μg/ml) for 24, 36, 48 and 72 h. Proliferation was measured with WST-1 reagent after the indicated period of time. Points represent the mean of three similar experiments (n=3); bars, SE.

rLz-8 caused a detectable accumulation of nuclei in G1 phase with a concomitant decrease of nuclei in S phase. This was most evident after 12, 18, 24 and 36 h (Fig. 1A). After 12, 18, 24 and 36 h of stimulation with 5 mg rLz-8, staining of cells of the gastric cell line SGC-7901 with DNA-specific fluorochrome propidium iodide (PI) showed 59.8, 61.7, 64 and 65.2% of SGC-7901 cells with G0/G1 DNA content, while 23.3, 22.8, 20.3 and 20.4% of cells were in S-phase, respectively.

Furthermore, treatment of SGC-7901 cells with rLz-8 for 24, 36, 48 and 72 h at different concentrations (0.5, 2.5, 5.0, 20, 50 μg/ml) also resulted in cell growth inhibition. Slight inhibition of proliferation was detected in cells exposed to 0.5 μg/ml of rLz-8, whereas cell proliferation was markedly inhibited in cells treated with 2.5 or 5 μg/ml. Treatment with 50 μg/ml rLz-8 for up to 72 h significantly reduced the number of viable human gastric cancer SGC-7901 cells (Fig. 1B). All results of the cell viability and proliferation assay WST-1 showed that rLz-8 inhibited SGC-7901 cell proliferation in a dose- and time-dependent manner, with IC_50_ at 24 h of 5 μg/ml.

### rLz-8 accumulating in nucleus triggers endoplasmic reticulum stress (ERS)-mediated cell death

**Figure 2:**
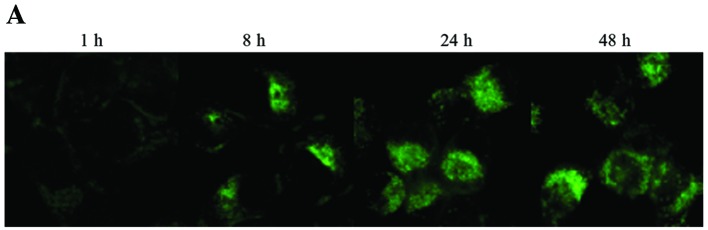

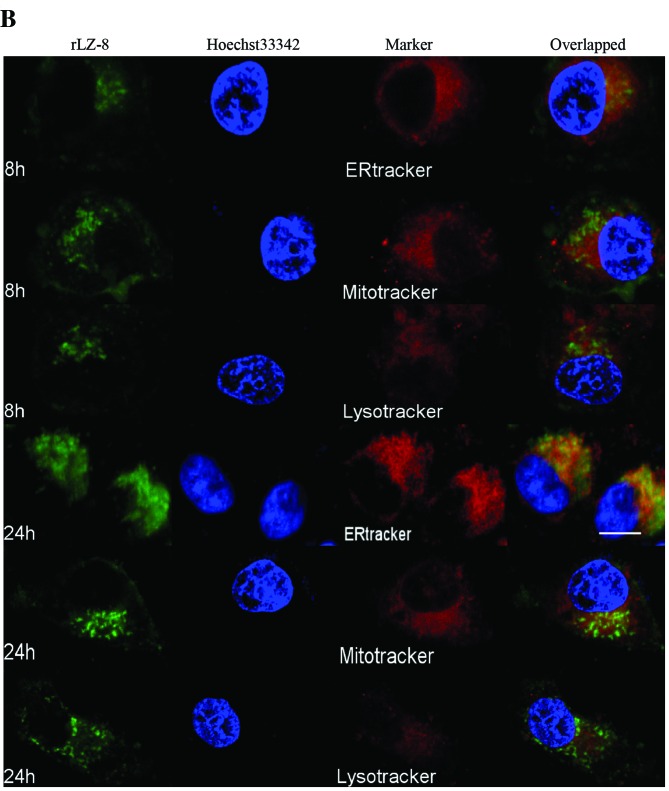

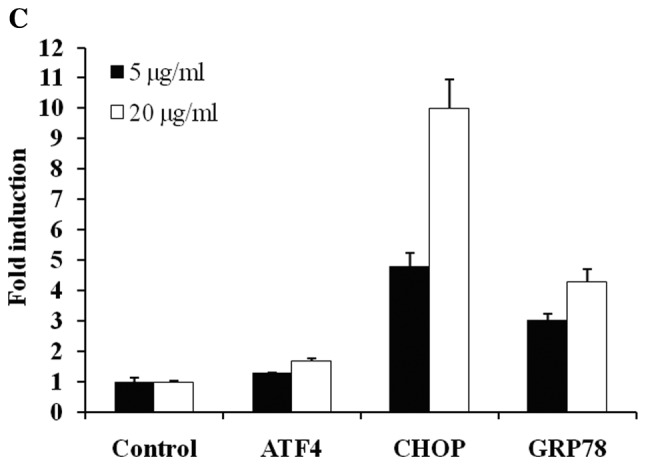

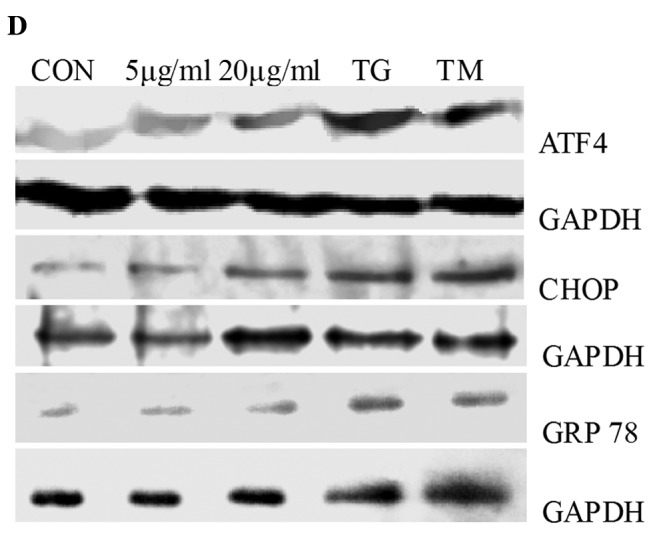

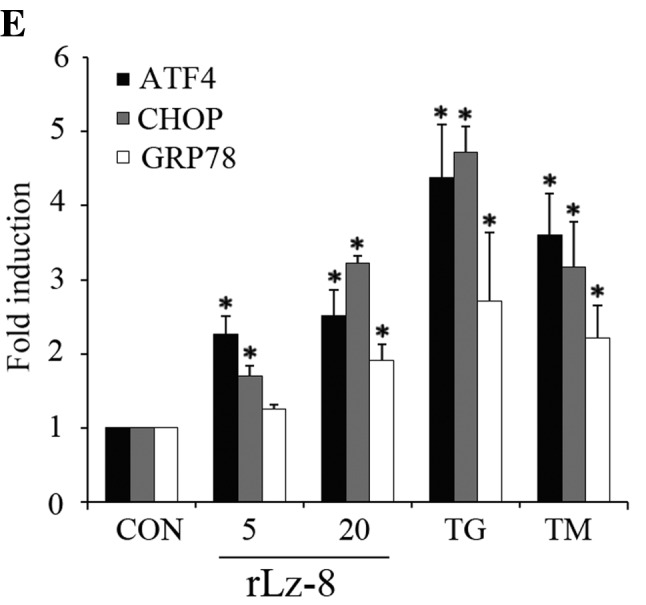
Localization of rLz-8 and induction of ER stress in cells treated with rLz-8. (A) rLz-8- FITC (5 μg/ml) accumulating in cells for several time courses. Scale bar: 10 μm. (B) Intracellular localization of rLz-8. SGC7901 cells treated with rLz-8-FITC for 24 h. After treatment, the rLz-8 localization was examined by using Hochest 33342 (1 μg/ml) for nucleus (blue) and various subcellular makers such as ERtracker (red) for ER, Mitotracker (red) for mitochondria, Lysotracker (red) for lysosome. Scale bar: 10 μm. (C) Gene expression of ER stress induced by rLz-8. SGC7901 cells treated with rLz-8 (5 or 20 μg/ml d) and incubated for 24 h. Relative mRNA levels were measured by real-time qPCR, n=3. (D) Protein levels of ATF4 (38 kDa), CHOP (30 kDa) and GRP78 (78 kDa) were measured by Western blot analysis. SGC7901 cells were treated for 24 h with 5 μg/ml rLz-8, 20 μg/ml rLz-8, 300 nM thapsigargin (TG), and 2 μg/ml tunicamycin (TM). (E) Quantitative analysis of intensities of Western blotting. CON, control. Standard error represents three independent experiments. P-value represents the significant difference between conditions at ^*^P<0.05.

The underlying molecular mechanism governing the effects of rLz-8 on the inhibition of SGC-7901 cell proliferation is still unclear. The first step in examining the possible roles of rLz-8 was to determine its cellular localization. Colocalization studies were performed using specific markers for the cellular organelles and rLz-8 in SGC-7901 cells. As shown in Fig. 2A and B, fluorescein isothiocyanate (FITC)-labeled rLz-8 and the ER's specific marker overlapped, suggesting that rLz-8 localized at the ER, into which it diffuses.

In the following studies, we examined whether rLz-8 triggered ER stress as it accumulated in the ER by determining the effects of rLz-8 on the expression of classical ER stress markers, such as the transcription factors CHOP/GADD153 and ATF4. When ER stress occurs, a large number of unfolded proteins accumulate in the ER, GRP78 dissociates from protein kinase RNA-like ER kinase (PERK) by binding these proteins. Unbound PERK oligomerizes and is activated by trans-autophosphorylation. Activated PERK phosphorylates eIF2α, then its phosphorylation promotes translation of ATF4, escalating ER stress further. ATF4 can function as a homodimer or heterodimer with members of the binding protein family of transcription factors, such as the cell death protein CHOP/GADD153, and also promotes translation of CHOP/GADD153 (18,19). Real-time quantitative RT-PCR (qRT-PCR) analyses showed that rLz-8 induced CHOP (4- to 9-fold), ATF4 (1- to 2-fold) and GRP78 (3- to 4-fold) mRNA 3- to 10-fold in SGC-7901 cells at 5 and 20 μg/ml (Fig. 2C), and the expression of ATF4 was confirmed by Western blotting (Fig. 2D). However, in the same test for expression of CHOP, we did not observe positive results. Leena *et al* (20) reported that some commercially available CHOP antibodies were non-specific for detecting CHOP in Western blotting and hypothesized that antibody specificity may influence the results.

**Figure 3:**
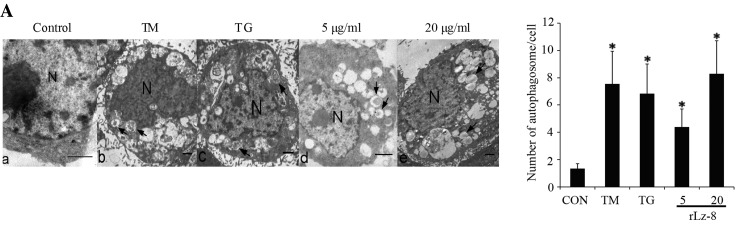

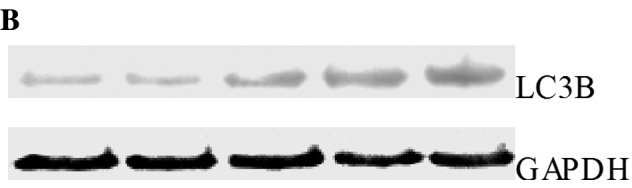

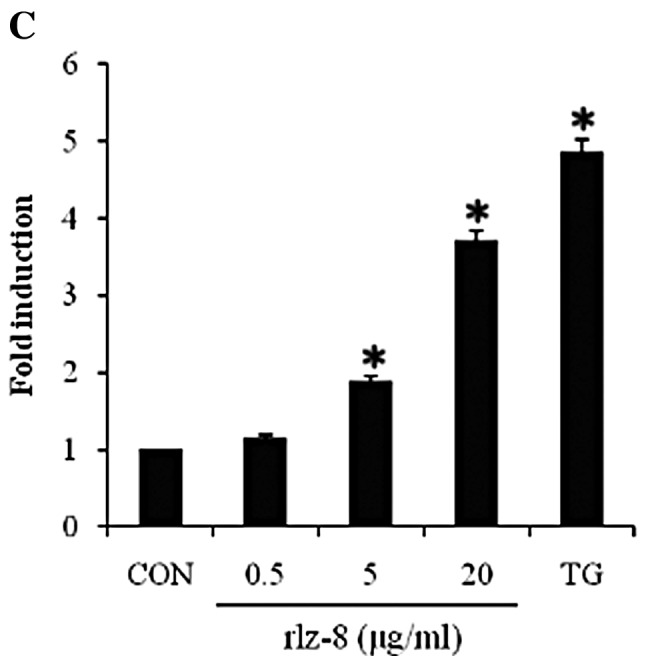

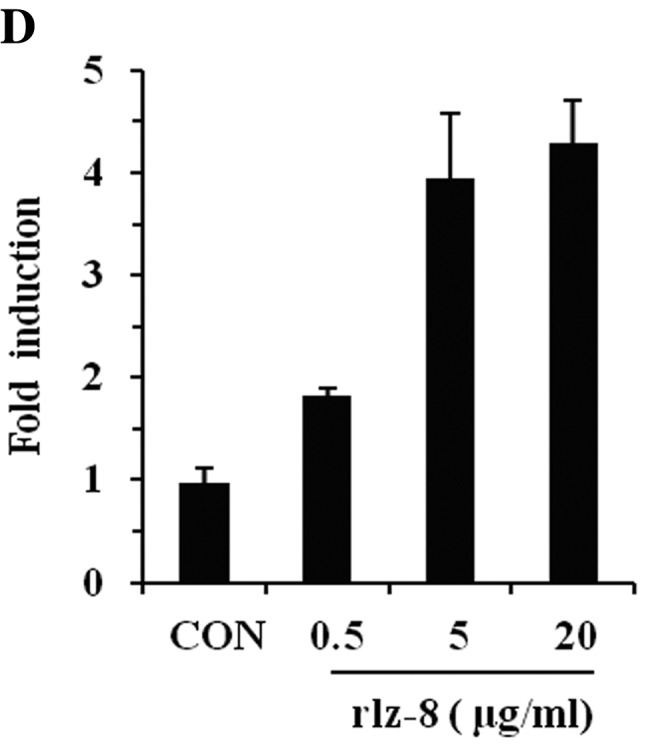

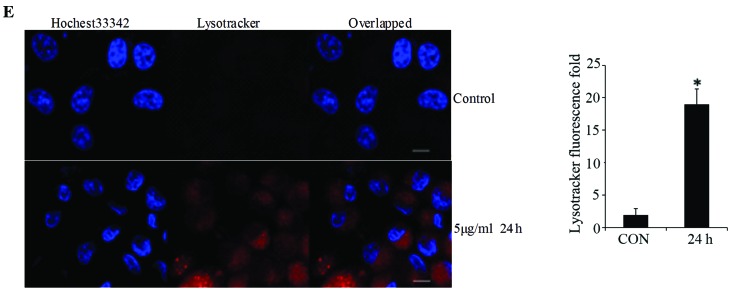

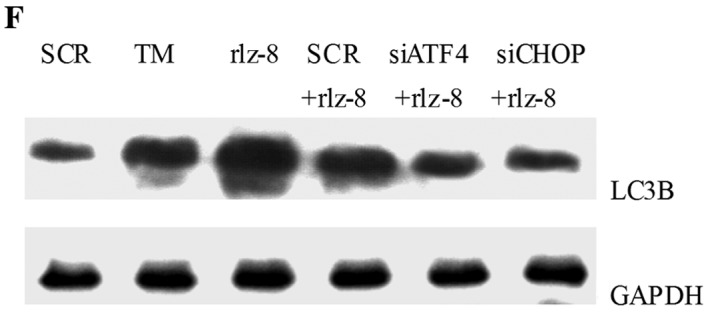

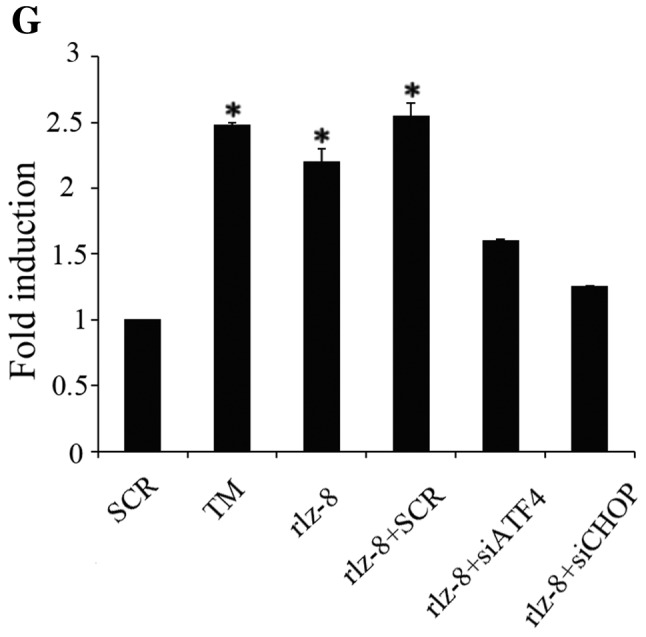

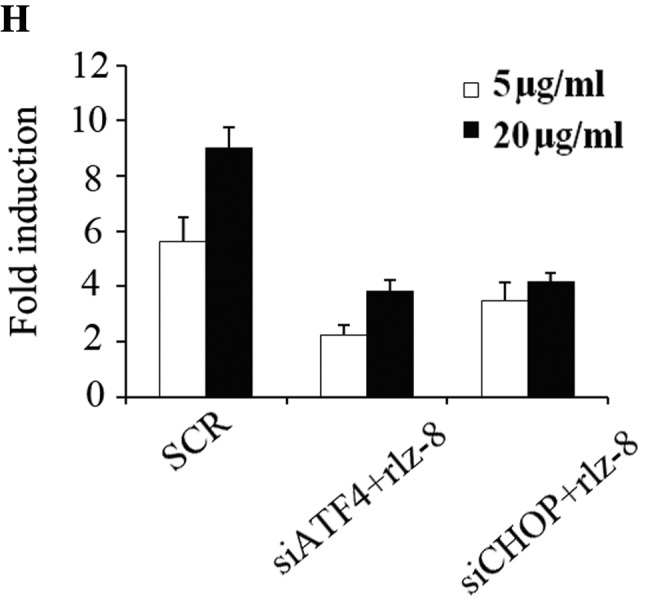
Induction of autophagy in SGC7901 cells treated with rLz-8. (A) Electron micrographs were taken of SGC7901 cells untreated (a) or treated (b) with 2 μg/ml tunicamycin (TM) for 24 h, (c) 300 nM thapsigargin (TG) for 24 h, (d) 5 μg/ml and (e) 20 μg/ml rLz-8 for 48 h. Double membrane vacuoles are denoted by an arrow, and the nucleus is denoted by N. Scale bar: 1 μm. The histogram is the numbers of autophagosomes in SGC7901 cells after exposure to the indicated stimuli for 24 h. A total of 25 electron microscopic sections were prepared. Standard error represents 3 independent experiments. P-value represents the significant difference between conditions at ^*^P<0.05. (B) Protein levels of LC3B (15 kDa) were measured by immunoblot analysis. SGC7901 cells were treated for 24 h with 300 nM thapsigargin (TG), 0.5, 5, and 20 μg/ml rLz-8. (C) Quantitative analysis of intensities of Western blotting. CON, control. Standard error represents 3 independent experiments. P-value represents the significant difference between conditions at ^*^P<0.05. (D) Gene expression of LC3B induced by rLZ-8. SGC7901 cells treated with 0.5, 5, and 20 μg/ml rLz-8 for 24 h. Relative mRNA levels were measured by real-time qPCR, n=3. CON, control. (E) Induction of lysosomes in SGC7901 cells treated with rLz-8. SGC7901 cells were treated with 5 μg/ml of rLz-8 for 24 h. Nuclear stained with Hoechst 33342 (blue) and Lysotracker (red) staining are shown. The histogram is quantitative analysis of fluorescence intensities. Scale bar: 10 μm. (F) Protein levels of LC3B (15 kDa) were measured by immunoblot analysis. Cells were RNAi against control (SCR), ATF4 and CHOP. After 24 h, cells were treated with 5 μg/ml rLz-8 for 24 h. (G) Quantitative analysis of intensities of Western blotting. CON, control. Standard error represents three independent experiments. P-value represents the significant difference between conditions at ^*^P<0.05. (H) Gene expression of LC3B with siRNA. Cells were RNAi against control (SCR), ATF4 and CHOP. Cells were treated 24 h later with 5 μg/ml or 20 μg/ml rLz-8 for 24 h. Relative mRNA levels were measured by real-time qPCR, n=3.

To determine the role of ER stress in the rLz-8-induced death of SGC-7901 cells, we transiently transfected cells with siRNA duplexes specific for CHOP and ATF4. Results showed that CHOP or ATF4 knockdown cells treated with rLz-8 had higher survival rates than the control cells (SCR; Fig. 3E). Therefore, rLz-8 serves as an inducer for death in SGC-7901 cells as it diffuses into the ER and triggers ER stress.

### The rLz-8-triggered ER stress induces an autophagic response in SGC-7901 cells

Considering the role of ATF4 in ER stress and autophagy, we next investigated whether rLz-8 induced an autophagic response in SGC-7901 cells. Electron microscopy remains one of the most accurate methods for the detection of autophagy and quantification of autophagic accumulation. Firstly, autophagic bodies and autophagosomes with double or single membrane structures in the cytoplasm were examined by electron microscopy for autophagic topical changes. In Fig. 3Aa, the electron micrograph depicts the intact cell and nuclear membrane, rough endoplasmic reticulum, distinct nucleus, and homogeneous cytoplasm of normal SGC7901 cells (control). SGC7901 cells treated with different doses of rLz-8 are shown in Fig. 3Ad and e. Membrane-bound autophagosomes in the cytoplasm are observed. The nucleus is condensed, but the nuclear membrane is intact and distinct. Tunicamycin (TM) and thapsigargin (TG) are triggers of the autophagic ER stress-induced response in cells. As shown in the electron micrograph in Fig. 3Ab and c, SGC7901 cells treated with TM and TG show an abnormally large increase in the number of autophagosomes, condensed nuclei, and damaged nuclear membranes. The number of autophagosomes was significantly increased in cells treated with these stimuli, compared to non-treated cells (Fig. 1B).

Microtubule-associated protein 1 light chain 3 (MAP1-LC3) is essential for autophagy and is associated with autophagosome membranes. MAP1-LC3 has two subunits, specifically MAP1-LC3A (MAP1-LC3α) and MAP1-LC3B (MAP1-LC3β) (21). There are two forms of LC3B, the cytosolic LC3-I and the membrane-bound LC3-II. During autophagy, LC3B is synthesized in large quantities. LC3-I is formed by the removal of the C-terminal 22 amino acids from newly synthesized LC3B, and a fraction of LC3-I is converted into LC3-II (22,23). Therefore, LC3B is thought to be an important marker for autophagy. During rLz-8 treatment, the protein and mRNA levels of LC3B increased significantly in a dose- and time-dependent manner (Fig. 3B and C), and there was an increase in the processed form of LC3B, which was indicative of increased autophagy. The result of real-time reverse-transcription PCR analysis showed that for the negative control (CON), positive control (TG) and rLz-8-treated groups (0.5, 5 and 20 μg/ml), mRNA levels of LC3B were up-regulated 1.8-, 3.9-, and 4.2-fold, respectively.

During rLz-8 treatment, the protein and mRNA levels of LC3, a recognized marker, increased significantly in a dose- and time-dependent manner (Fig. 3D), and there was an increase in the processed form of LC3. This was indicative of increased autophagy, particularly with 5 and 20 μg/ml, between the negative control (CON), positive control (TM, TG) and rLz-8-treated groups.

When autophagy is induced in old or unwanted organelles, proteins and microbes are delivered to the lysosome, accompanied by a rapid increase in the number of lysosomes. Lysotracker, a lysosome-specific dye, was used to examine the number of lysosomes in rLz-8-treated SGC-7901 cells. Results indicated that compared to the negative control group, cells treated with rLz-8 at 5 μg/ml for 24 h demonstrated a significant increase in lysosomal mass (Fig. 3E).

The relationship between autophagy and ER-stress was investigated by using siRNA duplexes specific for the ER-stress markers, CHOP and ATF. When CHOP and ATF4 were rendered dysfunctional, the LC3B protein and mRNA levels were greatly reduced compared to the control siRNA-transfected cells (Fig. 3F–H). Hence, we suggest that ER-stress is the main factor triggering the autophagy response.

### The role of autophagy in the death of SGC7901 cells treated with rLz-8

**Figure 4:**
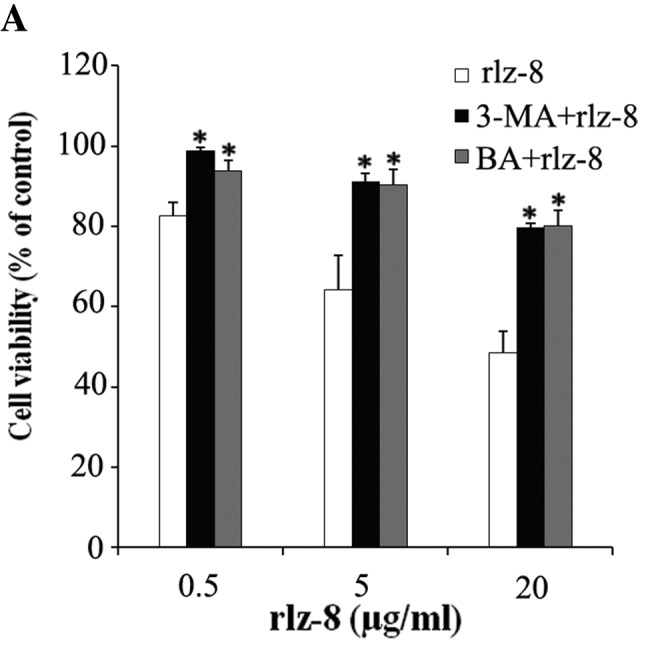

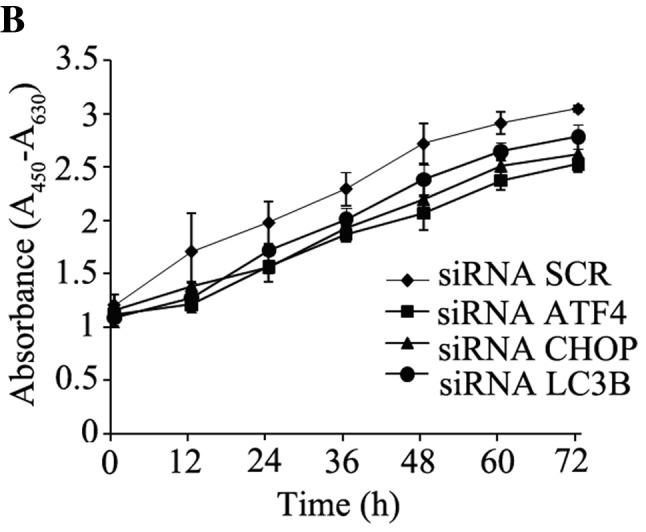

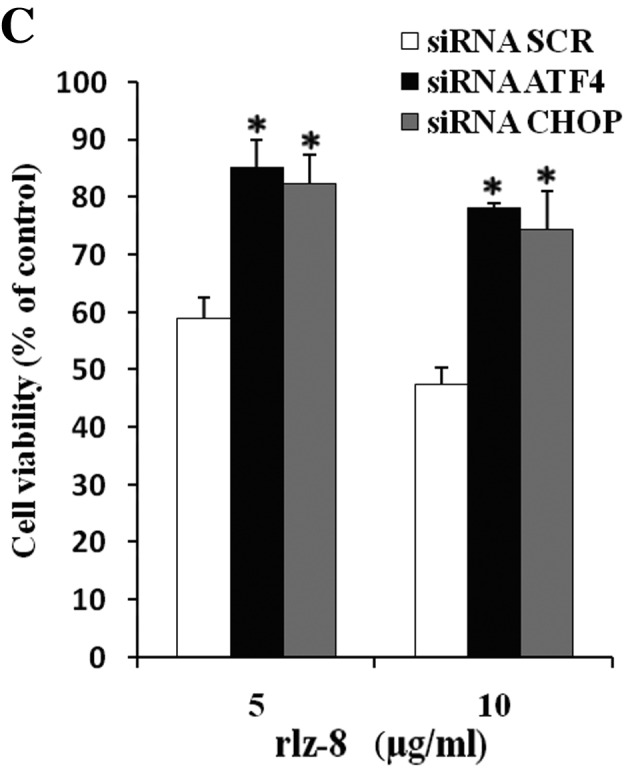

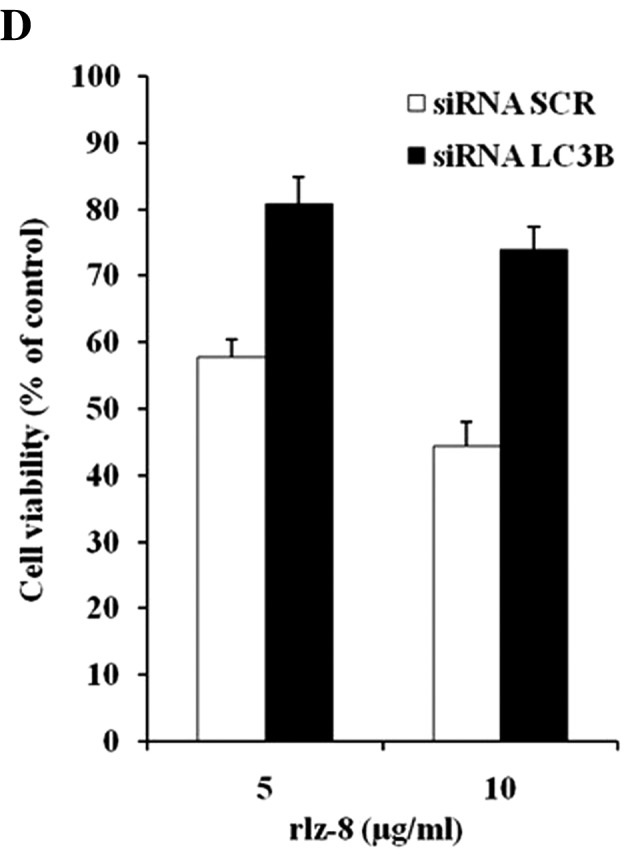

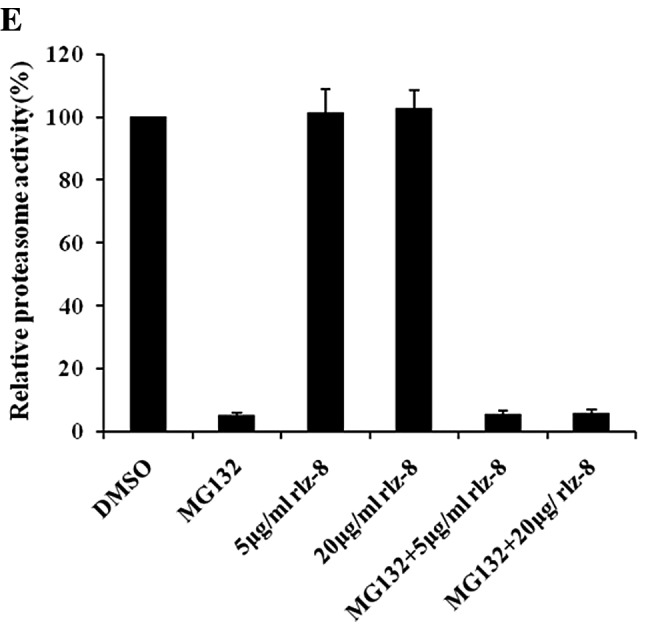
The role of autophagy in the death of SGC7901 cells treated with rLz-8. (A) Effect of rLz-8-induced autophagic cell death. SGC7901 cells non-treated or treated with 1 mM 3-MA or 30 μM BA for 1 h, and then treated with 0.5, 5 or 20 μg/ml rLz-8 for 24 h. Proliferation was measured with WST-1 reagent after the indicated time period. Standard error represents 3 independent experiments. P-value represents the significant difference between conditions at ^*^P<0.05. (B) Measurement of proliferation of SGC7901 cells in response to siRNA. Cells were transfected with siRNA against control (SCR), ATF4, and CHOP and siRNA LC3B. Six hours later, cells were cultured with DMEM supplemented with 10% FBS and WST-1 reagent was added after the indicated period of time. The absorbance was determined at the respective wavelength with an ELISA reader. Standard error represents 4 independent experiments. (C and D) Cell viability of SGC7901 cells treated with rLz-8. Cells were transfected with siRNA against control (SCR), ATF4, CHOP and siRNA LC3B. (E) Proteasome activity in SGC7901 cells treated with rLz-8. Proteasome activity in cells was directly measured using a cell-based assay after cells were treated with different combinations of drugs for 24 h. MG132 (100 nM) is a proteasome inhibitor. Standard error represents three independent experiments.

Over the years many studies have proven that autophagy plays a dual role in the biological process of cell death. To examine whether the autophagy induced by rLz-8 plays any roles in cell survival or death, cells in which autophagy was blocked by 3-methyladenine (3-MA; a specific inhibitor of autophagic/lysosomal protein degradation) were treated with rLz-8. A colorimetric WST-1 assay was used to determine the number of viable cells. Results showed that cell death that would otherwise be induced by rLz-8 was inhibited: the number of rLz-8-treated living cells was more than the control that was not treated with 3-MA (Fig. 4A). We next transiently transfected cells with siRNA duplexes specific for LC3B, then detected the survival rate of LC3B knockdown cells treated with rLz-8 for 24 or 48 h. Compared with the non-siRNA LC3B control in which expression of LC3B was inhibited, the rLz-8 killing effect declined rapidly (Fig. 4D). Hence, this suggests that autophagy plays a crucial role in the process of cell death induced by rLz-8.

### Caspase inhibitors do not prevent rLz-8-induced cell death

**Figure 5:**
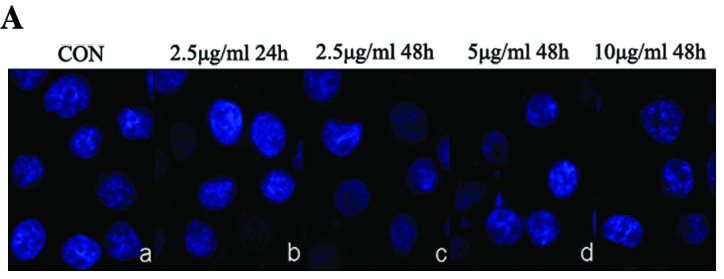

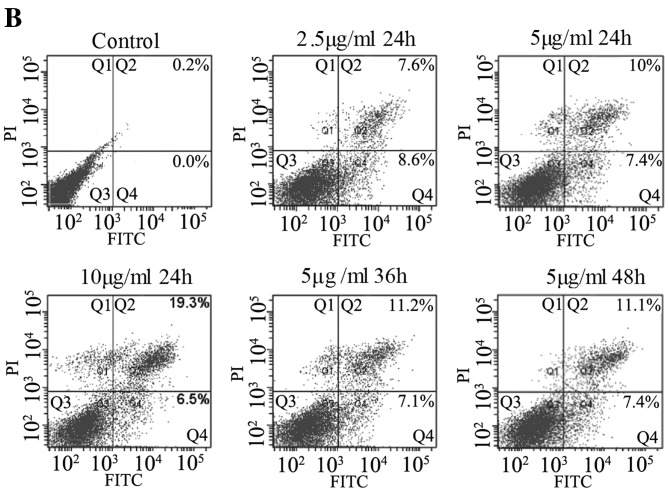

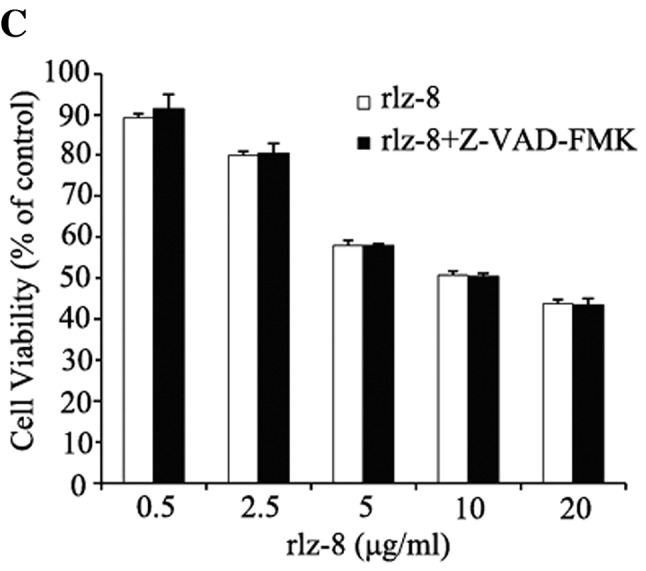

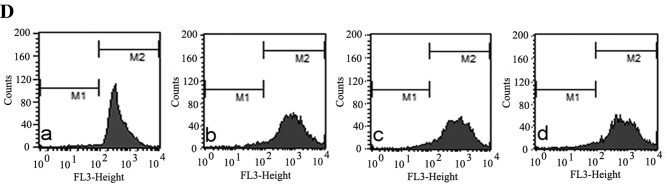
(A) Morphological changes of nuclear in SGC7901 cells treated with rLz-8. SGC7901 cells untreated (a) or treated (b–e) with 2.5, 5, or 10 μg/ml rLz-8 for several time courses (24 h, 48 h), and then 1 μg/ml Hoechst 33342 stained nuclear for 20 min. (B) Effect of rLz-8 on cell viability of SGC7901 cells. AV/PI double staining was performed on SGC7901 cells before and exposure to 2.5, 5, and 10 μg/ml rLz-8 for different periods of time (24, 36 and 48 h). AV^-^/PI^+^(Q1): percentage early necrotic cells; AV^+^/PI^+^(Q2): percentage late apoptotic and dead cells, AV^−^/PI^−^(Q3): percentage viable cells, AV^+^/PI^−^(Q4): percentage early apoptotic cells. (C) Effect of caspases in SGC7901 cells treated with rLz-8. SGC7901 cells untreated or treated with 50 μm Z-VAD-FMK for 30 min, and then treated with 0.5 to 20 μg/ml rLz-8 for 48 h. The result measured with WST1. Standard error represents 4 independent experiments. (D) Effect of rLz-8 on mitochondrial membrane potential of SGC7901 cells. SGC7901 cells untreated (a) or treated (b–d) with 0.5, 5, and 20 μg/ml rLz-8 for 24 h, and then cells were incubated with 10 μg/ml JC-1 for 30 min.

When apoptosis occurs, significant morphological changes such as chromatin condensation, nuclear shrinkage, and the disappearance of the nuclear envelope and nucleolus may be seen in cells. To investigate the morphological changes of organelles in rLz-8-treated SGC-7901 cells, laser scanning confocal microscopy was used to analyze Hochest 33342-stained nuclei. As shown in Fig. 2A, normal cells have a beam-shaped nucleus, smooth nuclear envelope, and non-condensed zones of chromatin. The cancer cell line SGC-7901 was treated with rLz-8 for 24 and 48 h at concentrations ranging from 2.5 to 10 μg/ml. Compared with normal cells, no apoptotic morphological changes were seen in any of the rLz-8-treated groups (Fig. 2A). Most significantly, in the high-dose rLz-8 treatment group (10 μg/ml), nearly all cells were dead, but we did not observe, by transmission electron microscopy (TEM), any apoptotic morphological changes that had occurred during the process of death (Fig. 3A). We also analyzed the apoptosis rate of the treatment groups by using flow cytometry (Fig. 5B), but results indicated that rLz-8 could not induce apoptosis in SGC-7901 cancer cells in a time- and dose-dependent manner.

Although it is accepted that apoptosis is mediated by caspase activity, the specific role of caspases is debatable (17,24–29). A caspase inhibition assay was performed to examine the involvement of caspase in rLz-8-induced cell death. The caspase inhibitor z-VAD-fmk (50 mM) did not significantly affect the cell viability of rLz-8-treated SGC-7901 cells (Fig. 5C). These results proved that rLz-8-induced cell death is independent of caspase activation. It is well known that when apoptosis occurs, a change in the electric potential of the mitochondrial membrane is a typical event. Herein, flow cytometry was used to investigate changes in mitochondrial membrane potential in rLz-8-treated SGC7901 cells. These data together with the siRNA assay results corroborate that rLz-8-induced cell death occurs by autophagy.

## Discussion

Disturbances in normal functions of the ER lead to the UPR, an evolutionarily conserved cell stress response which is aimed initially at compensating for damage but can eventually trigger cell death if ER dysfunction is severe or prolonged. The mechanisms by which ER stress leads to cell death remain enigmatic, with multiple potential participants described with little clarity, with specific death effectors dominating in particular cellular contexts (30).

As we know, types of cell death induced by ER stress include apoptotic cell death, necrosis, and autophagic cell death (31). Among these, apoptotic cell death depends greatly on caspase activity. Many important relevant mechanisms involved in caspase-dependent cell death have already been studied extensively (32).

In our study, rLz-8 exerted a promising killing effect on SGC-7901 cells. We used classical methods to examine whether caspase-independent cell death rather than caspase-dependent apoptosis was triggered by rLz-8-induced ER stress. The results of TEM indicated that no morphological feature of caspase-dependent apoptosis was observed in rLz-8-treated cells, i.e., chromatin condensation, loss of cytoplasmic processes, round cell shape or fading of the nuclear membrane (33–36). Moreover, when caspase families were inhibited with the pan-caspase inhibitor z-VAD-fmk in SGC-7901 cells, we found that rLz-8 still had the same killing effect, affirming that rLz-8-induced cell death is not caspase-dependent, and may not even be apoptosis (37–41).

Regarding rLz-8-induced cell death, our findings suggest: 1) In SGC-7901 cells, rLz-8 diffuses and accumulates gradually in the ER, reaching saturation after 24 h. rLz-8 was not transported out of the ER or degraded until the cells died. 2) After rLz-8 accumulated in the ER, a significant increase in the number of lysosomes was observed, by fluorescence microscopy. The results of TEM also confirmed dilated and vacuolized smooth ER and an increased number of lysosomes. 3) rLz-8-treated cells showed increased mRNA and protein levels of CHOP/GADD153 and ATF4, respectively. 4) When rLz-8 gradually accumulated in the ER of SGC-7901 cells, autophagy and cell death were observed. When autophagy was inhibited by 3-MA treatment, the number of dead cells decreased, suggesting that autophagy played a role in promoting cell death in rLz-8 treated cells.

Therefore, how does the cell deal with the aggregation of rLz-8 in the ER? Excess amounts of unfolded or misfolded proteins in the ER are retrotranslocated to the cytoplasm and degraded by the ERAD system, which includes a ubiquitin/proteasome and an autophagy/lysosome system (15). A larger number of autophagosomes and lysosomes appeared in rLz-8-treated cells. This suggests that the autophagy/lysosome system was activated, and the cell may employ this system to degrade rLz-8. In the study of Fujita *et al* (5), native and mutant dysferlin were degraded by the ubiquitin/proteasome ERAD system. However, when mutant dysferlin was not sufficiently degraded by ubiquitin/proteasome ERAD system, it aggregated in the ER and stimulated autophagy formation and LC3 conversion via the ER stress-related PERK-eIF2a-ATF4-CHOP/GADD153 pathway. This pathway was also activated in rLz-8-treated cells. The mutant dysferlin aggregates on the ER membrane could be recognized by the elongating membrane that forms the autophagosome, and is then degraded by the autolysosome upon fusion with a lysosome. Although there were no experimental results in our study verifying that rLz-8 was recognized and degraded by the ubiquitin/proteasome ERAD system, we used a Bayesian discriminant method (42,43) to predict the rLz-8 ubiquitination sites. However, specific ubiquitination sites were not found.

In general, the ERAD system degrades aberrant proteins to ensure cell survival, but in this study the SGC-7901 cells were killed by the accumulation of rLz-8 in the ER. This is similar to the apoptotic death of C2C5 cells caused by dysferlin aggregates, or protein overexpressing in yeast. It is known that excessive ER stress, or ER-stress mediated cell death, can kill cells but what are the roles of ERAD and autophagy in this process? According to a recent study, autophagy formation was a cellular defense mechanism against polyQ72-induced ER-stress-mediated cell death, whose mechanism involved the degradation of polyQ72 aggregates (44). Similar studies also indicated that autophagy protects cells from ER stress arising from protein aggregations via the ERAD system (45,46). However, we have determined that while aggregations of rLz-8 induced ER stress and triggered the ERAD system, the resulting autophagy contributed to cell death rather than survival. Before this, it was very rare that a heterologous protein aggregating in the ER of a tumor cell could trigger autophagy-dependent cell death.

Next, what role did ER stress play in the rLz-8-induced cell death? As we know, when ER stress exceeds a threshold it mediates many types of cell death, including classic apoptosis, autophagic cell death and necrosis. The principal challenge with any cellular strategy for preventing death caused by ER stress lies with the multitude of parallel pathways that potentially lead to downstream cell death mechanisms. Multiple pathways linking ER stress to cell death have been reported. Of these, ATF4-CHOP/GADD153 is the first identified protein that mediates ER stress-induced apoptosis and much is known of the roles of this molecule in cell death. Nevertheless, little is known of just how ATF4-CHOP/GADD153 induces cell death, especially considering that CHOP-deficient cells are resistant to ER stress-mediated cell death. Our study showed that when expression of ATF4 or CHOP was inhibited in rLz-8-treated SGC-7901 cells, dead cells decreased significantly, and LC-3II conversion was down-regulated. This proves that the ATF4-CHOP pathway and ER-stress both are the key factors for inducing cell death, and also that the ATF4-CHOP pathway acts as a bridge between ER stress and autophagy.

In conclusion, rLz-8 aggregation induces autophagic cell death and provides a novel mechanism for protein-based anti-cancer medicine. This may lead to new strategies to develop therapeutic drugs that will target cancer cells to undergo autophagic cell death independent of apoptosis.
